# Frequency and Pathophysiology of Acute Liver Failure in Ornithine Transcarbamylase Deficiency (OTCD)

**DOI:** 10.1371/journal.pone.0153358

**Published:** 2016-04-12

**Authors:** Alexander Laemmle, Renata C. Gallagher, Adrian Keogh, Tamar Stricker, Matthias Gautschi, Jean-Marc Nuoffer, Matthias R. Baumgartner, Johannes Häberle

**Affiliations:** 1 Division of Metabolism and Children`s Research Center (CRC), University Children`s Hospital, Zurich, Switzerland; 2 radiz–Rare Disease Initiative Zurich, Clinical Research Priority Program for Rare Diseases, University of Zurich, Zurich, Switzerland; 3 Department of Pediatrics, Medical Genetics, University of California San Francisco, San Francisco, United States of America; 4 Department of Clinical Research and Clinic for Visceral Surgery and Medicine, Bern University Hospital, Bern, Switzerland; 5 Department of Pediatrics, University Children's Hospital, Bern, Switzerland; 6 University Institute of Clinical Chemistry, University of Bern, Bern, Switzerland; 7 Zurich Center for Integrative Human Physiology, University of Zurich, Zurich, Switzerland; National Institutes of Health, UNITED STATES

## Abstract

**Background:**

Acute liver failure (ALF) has been reported in ornithine transcarbamylase deficiency (OTCD) and other urea cycle disorders (UCD). The frequency of ALF in OTCD is not well-defined and the pathogenesis is not known.

**Aim:**

To evaluate the prevalence of ALF in OTCD, we analyzed the Swiss patient cohort. Laboratory data from 37 individuals, 27 females and 10 males, diagnosed between 12/1991 and 03/2015, were reviewed for evidence of ALF. In parallel, we performed cell culture studies using human primary hepatocytes from a single patient treated with ammonium chloride in order to investigate the inhibitory potential of ammonia on hepatic protein synthesis.

**Results:**

More than 50% of Swiss patients with OTCD had liver involvement with ALF at least once in the course of disease. Elevated levels of ammonia often correlated with (laboratory) coagulopathy as reflected by increased values for international normalized ratio (INR) and low levels of hepatic coagulation factors which did not respond to vitamin K. In contrast, liver transaminases remained normal in several cases despite massive hyperammonemia and liver involvement as assessed by pathological INR values. In our *in vitro* studies, treatment of human primary hepatocytes with ammonium chloride for 48 hours resulted in a reduction of albumin synthesis and secretion by approximately 40%.

**Conclusion:**

In conclusion, ALF is a common complication of OTCD, which may not always lead to severe symptoms and may therefore be underdiagnosed. Cell culture experiments suggest an ammonia-induced inhibition of hepatic protein synthesis, thus providing a possible pathophysiological explanation for hyperammonemia-associated ALF.

## Introduction

**Fig 1:**
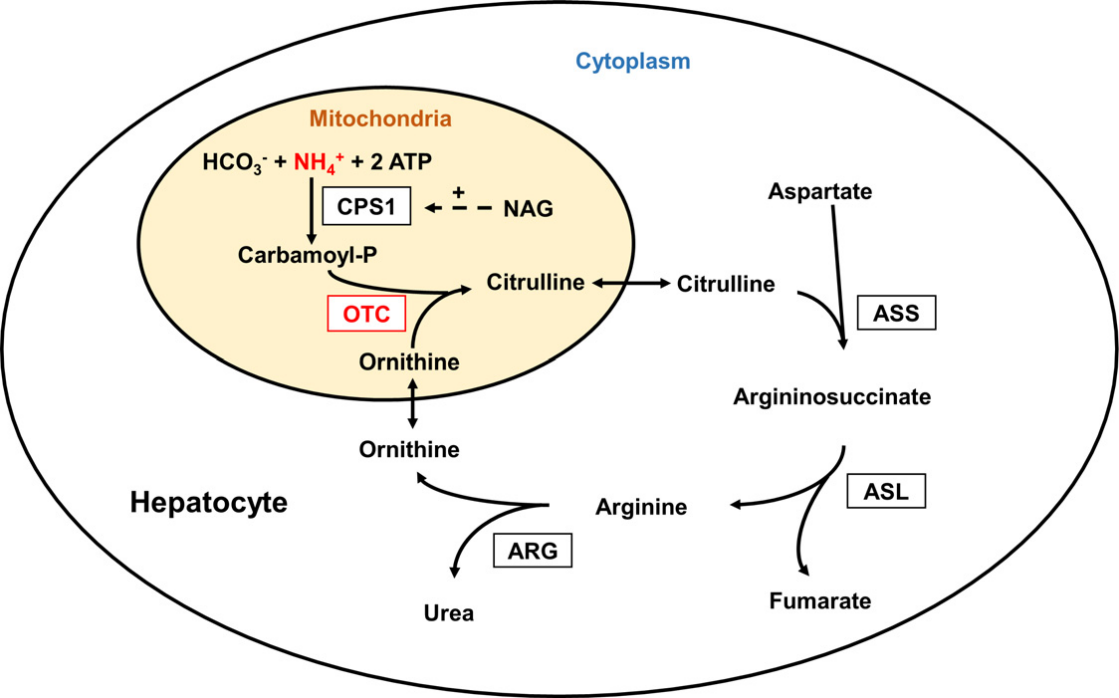
Urea cycle. In the urea cycle the “toxin” ammonium (NH_4_^+^) is converted to non-toxic urea by five consecutive enzymatic reactions in the liver. In hepatocytes, the rate-limiting, ATP-dependent enzyme carbamoyl phosphate synthetase 1 (CPS1), which is allosterically activated by N-acetyl glutamate (NAG), produced by N-acetyl glutamate synthase (NAGS), not shown, and ornithine transcarbamylase (OTC) are located in the mitochondria, while argininosuccinate synthetase (ASS), argininosuccinate lyase (ASL) and arginase (ARG) are in the cytoplasm. Inherited defects in any of these enzymes can cause recurrent episodes of hyperammonemia. Defects in two mitochondrial transporters, not shown, may also result in hyperammonemia.

Patients with inherited defects in the urea cycle (Fig 1) are at risk of recurrent acute metabolic crises with life-threatening hyperammonemia, associated with acute or chronic brain damage leading to severe neurological long-term disabilities, impairing quality of life and leading to significant mortality [1–4]. The main trigger for metabolic crises is an imbalance between a reduced caloric uptake and an increased caloric demand causing protein catabolism and hence provoking hyperammonemia.

In addition to neurological complications, patients with acute hyperammonemia may suffer from recurrent episodes of acute liver failure (recurrent ALF, RALF) with associated coagulopathy [5–9]. In the largest hitherto published study, reporting 89 individuals with ornithine transcarbamylase deficiency (OTCD, MIM #311250) evaluated at two large metabolic centers (Children`s Hospital Colorado and the University of California, Los Angeles Medical Center), 57% of 49 symptomatic patients had liver involvement (liver injury as determined by elevated liver transaminases and/or ALF as determined by pathological coagulation parameters) [6]. However, the pathophysiology of this complication is poorly understood [3, 6, 10, 11].

In severe cases, ALF may require urgent liver transplantation, which is essentially curative of OTCD, however, there are several limitations including liver organ shortage, short and long-term morbidity and mortality including lifelong immunosuppressive therapy with major associated complications. In neonates, urgent liver transplantation is often not a feasible therapeutic option due to practical restrictions associated with small size.

In ALF associated with metabolic diseases, there is a lack of consensus criteria for liver transplantation [12]. Some UCD patients have undergone liver transplantation due to the severe impairment of liver function during their first metabolic crisis, and before the diagnosis was definitively confirmed [9]. However, other patients completely recovered despite a similar severity of liver failure [13, 14]. It was thus suggested that before liver transplantation is performed in patients with ALF of unknown cause, results of a full metabolic screening should be awaited [5, 8, 15]. Liver histology does not help to stratify patients, as it was grossly unremarkable in UCD patients suffering from ALF, only occasionally displaying subtle structural changes even in older patients [16]. Thus, the clinical and histological manifestation of liver disease in UCD patients is distinct from patients with chronic hepatitis, chronic liver failure or cirrhosis. ALF is also observed in patients with other metabolic diseases, for instance in pyruvate dehydrogenase (PDH) complex deficiency [17], which can present with secondary hyperammonemia.

The role of ammonia toxicity in contributing to the development of ALF was recently emphasised [18]. In contrast to adult patients with e.g. liver cirrhosis, in which elevated ammonia levels are the consequence of (chronic) liver failure, we hypothesise that hyperammonemia is a relevant trigger of ALF in UCD patients.

To substantiate this hypothesis, we analysed events of ALF in the Swiss OTCD patient cohort. In addition, we exposed human primary hepatocytes to ammonium chloride (NH_4_Cl) to find evidence for a causative role of hyperammonemia in impairing hepatic protein synthesis.

## Methods and Materials

### Study cohort

For the present study, we analysed the Swiss cohort of OTCD patients. All subjects were diagnosed and treated between 12/1991 and 03/2015 in a Swiss metabolic center. Vitamin K was not administered systematically, however some patients received it according to their coagulation status based on the decision of the treating center. The majority of patients was enrolled in the National Institutes of Health (NIH)-funded, multi-site Longitudinal Study of the Urea Cycle Disorders Consortium (UCDC) [19, 20] in which the University Children`s Hospital Zurich has been a partner since 09/2008. Written informed consent was obtained from each patient or their legal guardian(s), and the responsible ethics committee approved this natural history study (Kantonale Ethikkommission; StV 39/07).

### Clinical and laboratory data collection

From each subject with (biochemically or molecular genetically) confirmed OTCD, a detailed medical history was taken including family history, age and clinical symptoms at presentation and at diagnosis, and all available medical records and laboratory data from the past were obtained for review. Patients are usually seen twice a year (adults only once a year) for regular study visits. These include a detailed history of metabolic decompensations and hospitalizations, as well as a physical examination and laboratory evaluation. According to the study protocol, laboratory values to assess metabolic status (plasma ammonia, plasma amino acid profile and urinary orotic acid), liver function (various liver parameters including aspartate aminotransferase (ASAT), alanine aminotransferase (ALAT) and albumin) and coagulation (INR/prothrombin time (PT)/Quick percentage value, activated partial thromboplastin time (aPTT) and fibrinogen) were taken. Response to vitamin K was evaluated in some of the patients. From patients who suffered from RALF, either the crisis with the most pathological INR/PT or Quick percentage value was chosen or a representative episode where most of the laboratory values were available.

For this study, we used a simple definition of ALF, which is, however, based on and in full agreement with the one used by the “Pediatric Acute Liver Failure Study Group” in which an INR ≥1.5 together with clinical signs of encephalopathy is considered sufficient to define ALF [21, 22]. Thus, since all of our patients with an INR ≥1.5 showed some neurological signs (although in very rare cases only soft clinical signs and not distinguishable whether chronic or related to the acute situation), we did not add any further criteria to define ALF (e.g. biochemical evidence of acute liver injury). In a few cases for which no INR values were available, we analysed Quick percentage values or PT.

### Culture and NH_4_Cl treatment of primary human hepatocytes

Primary human hepatocytes were isolated from resected liver tissue from a non-affected (regarding OTCD) male patient undergoing liver surgery after having obtained informed consent. Hepatocytes were cultured according to an established standard procedure [23] and 6.0x10^4^ cells/cm^2^ were seeded on collagen-coated plastic dishes (96-well-plates for cell viability assays and 60 mm dishes for all other experiments) prior to culture in Dulbecco`s minimum essential medium (DMEM) supplemented with 10% fetal bovine serum, 50 U/mL penicillin, 50 μg/mL streptomycin, and 1 μmol/L dexamethasone. After overnight culture, the medium was replaced by serum-free Williams E medium®glutaMax™, which contains the dipeptide L-alanyl-L-glutamine, a more stable form of L-glutamine, thus preventing its deamination and additional (unwanted) release of ammonia. Twenty-four hours after serum deprivation, hepatocytes were treated with different concentrations of NH_4_Cl (0, 0.1, 1 and 10 mmol/L) for 24 and 48 hours in the same serum-free Williams E medium®glutaMax™.

### Cell viability assay

After 24 and 48 hours of NH_4_Cl treatment, cell viability was assessed by [3-(4,5-dimethyl-thiazol-2-yl) 2,5-diphenyltetrazolium bromide] MTT assay (Life Technologies™) according to the manufacturer`s protocol.

### Albumin quantification

After 24 and 48 hours of NH_4_Cl treatment, cell culture supernatant was collected in an Eppendorf tube and centrifuged at 10`000 g for 5 minutes. Supernatant was then transferred to a fresh Eppendorf tube and stored at -80°C until analysis. Albumin production and secretion was quantified by ELISA (Bethyl Laboratories Inc.) according to the manufacturer`s protocol.

### Quantification of urea and ASAT

Supernatant samples were also used for the quantification of urea production. Urea was quantified by cation exchange chromatography using the Biochrom 30+ Amino Acid Analysis System (Analytical® Technologies Limited) according to the manufacturer`s protocol.

The same supernatant samples were further analyzed for the quantification of ASAT levels which were determined in the routine clinical chemistry laboratory with the Beckman Coulter Synchron® System according to the manufacturer`s protocol.

### Statistical analysis

All statistics were performed applying Graphpad Prism 6 software. For statistical analysis of cell culture studies Student’s T-test was applied and p < 0.05 considered significant. Correlation studies between plasma ammonia levels and INR values in patients were performed by calculating the Pearson`s correlation coefficient and significance was tested by the two-tailed test and p < 0.05 considered as significant. For this purpose only patients with at least 5 simultaneously obtained ammonia and INR values were included.

## Results

### Study cohort

The characteristics of the Swiss OTCD cohort are listed in Table 1. The 37 patients (age 4–50 years in September 2015) derive from 21 families. Of 27 females (mean age at diagnosis 17.5 years, range 10 months-41 years), 19 are symptomatic (including two deaths during the initial metabolic crisis), five are asymptomatic, and information is lacking from three patients. From the ten male patients (mean age at diagnosis 4 months, range 3 days-24 months), seven suffered from a neonatal onset of disease that was fatal in five patients. Two males (No. 4.1 and 6.1) received liver transplantation at the ages of 4 and 15 years, respectively.

**Table 1:** Summary of the Swiss cohort of OTC deficient patients.

*No*.^*a*^	*Sex*^*b*^	*Age at Diagnosis*^*c*^	*Current Age*^*d*^	*Symptoms*^*e*^	*Mutation (NM_000531*.*5)*^*f*^	*References*^*g*^
1	f	18 m	25 y	yes	no mutation found	
2	f	2 y	24 y	yes	c.421C>T; p.Arg141*	[24]
3	f	19 m	20 y	yes	no mutation found	
4.1	m	2 m	17 y	yes	c.540G>C; p.Gln180His	[25]
4.2	f	33 y	46 y	no	c.540G>C; p.Gln180His	
4.3	f	15 y	28 y	no	c.540G>C; p.Gln180His	^ ^
5	f	13 y	30 y	yes	c.533C>T; p.Thr178Met	[26]
6.1	m	7 d	16 y	yes	c.386G>A; p.Arg129His	[27]
6.2	f	34 y	50 y	no	c.386G>A; p.Arg129His	^ ^
7.1	f	24 y	38 y	yes	c.506C>A; p.Pro169His	this study
7.2	f	prenatal	7 y	yes	c.506C>A; p.Pro169His	^ ^
8.1	m	7 d	†	death	c.717G>A; p.Glu239 =	[28]
8.2	f	34 y	48 y	yes	c.717G>A; p.Glu239 =	
9	f	10 m	12 y	yes	no mutation found	
10	f	7 y	18 y	yes	c.583G>A; p.Gly195Arg	[29]
11	f	20 m	10 y	yes	c.674C>T; p.Pro225Leu	[30]
12	f	8 y	17 y	yes	c.422G>A; p.Arg141Gln	[31]
13.1	f	16 y	†	death	c.538C>T; p.Gln180*	this study
13.2	f	38 y	38 y	yes	c.538C>T; p.Gln180*	
13.3	f	n.a.	n.a.	n.a.	c.538C>T; p.Gln180*	
13.4	f	n.a.	n.a.	n.a.	c.538C>T; p.Gln180*	
13.5	f	n.a.	n.a.	n.a.	c.538C>T; p.Gln180*	
13.6	m	prenatal	†	death	c.538C>T; p.Gln180*	
14.1	m	9 d	8 y	yes	c.386G>A; p.Arg129His	[27]
14.2	f	3 y	11 y	yes	c.386G>A; p.Arg129His	
14.3	f	41 y	50 y	no	c.386G>A; p.Arg129His	
15	f	6 y	†	death	c.274C>T; p.Arg92*	[32]
16.1	m	11 m	6 y	yes	c.386G>A; p.Arg129His	[27]
16.2	f	31 y	36 y	no	c.386G>A; p.Arg129His	
17.1	m	4 d	†	death	c.584G>C; p.Gly195Ala	this study
17.2	f	26 y	31 y	yes	c.584G>C; p.Gly195Ala	
18	m	3 d	†	death	c.548A>G; p.Tyr183Cys	[33]
19.1	m	2 y	4 y	yes	c.860C>T; p.Thr287Ile	this study
19.2	f	36 y	38 y	yes	c.860C>T; p.Thr287Ile	
20	f	2 y	4 y	yes	c.274C>T; p.Arg92*	[32]
21.1	m	3 d	†	death	c.674C>T; p.Pro225Leu	[30]
21.2	f	28 y	29 y	yes	c.674C>T; p.Pro225Leu	

^**a**^No., case number, in families with more than one case, x.1 stands for the index case which was diagnosed by selective screening in all 21 families

^**b**^Sex; f, female; m, male

^**c**^Age at diagnosis (in most cases diagnosis was first established biochemically and later confirmed by mutational analysis) and

^**d**^Current age (as of September 2015); d, days, m, months, y, years

†, deceased; n.a., not available

^**e**^Symptoms; no = no symptoms; yes = any symptoms from mild to severe; death = deceased due to (complications of) OTCD

^**f**^Mutations are indicated by changes in the coding DNA (c.) and protein (p.) reference sequences (NM_000531.5 and NP_000522.3, respectively) following the Human Genome Variation Society nomenclature.

^**g**^Previously described mutations are cited in the reference list, four mutations are novel.

All 21 index patients were diagnosed by selective screening, and 16 patients were diagnosed due to affected family members (two patients were diagnosed prenatally).

Mutational analysis in our cohort revealed 14 different *OTC* mutations of which four are novel changes. In three females, no mutation was found and diagnosis was based on the typical biochemical profile including orotic aciduria. The most prevalent mutation in our cohort, the well-characterized change c.386G>A; p.Arg129His [34, 35], was found in seven patients from three families.

### Acute liver failure in study cohort

**Fig 2:**
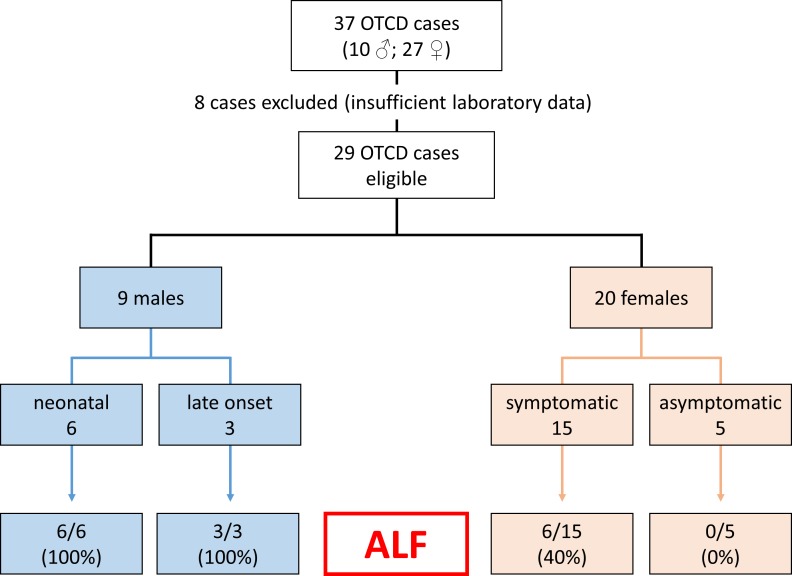
Overview of included patients and occurrence of acute liver failure. From 37 cases, 29 were eligible for further studies regarding occurrence of acute liver failure (ALF). Male patients are shown as neonatal (onset in the first month of life) and late onset (symptoms beyond the first month of life) cases. Females are classified as symptomatic or asymptomatic. The bottom boxes indicate the occurrence of ALF.

Of the 37 patients, 29 could be analyzed regarding ALF (Fig 2) (9 males and 20 females), while one male and seven female cases were excluded from all further studies due to a lack of clinical and/or laboratory data (missing medical reports and no values for coagulation tests). 6/9 male patients had a neonatal onset of OTCD with ALF. 4/6 neonatal male patients deceased during the initial crisis, and the two who survived the neonatal period experienced RALF. All late onset male patients suffered from ALF (RALF in 2/3 and a single episode in 1/3). Thus, all nine male patients suffered from ALF that was recurrent in 4/5 alive patients.

15/20 females were symptomatic, of whom six had documented episodes of ALF (recurrent in 2/6). None of the five asymptomatic females experienced ALF. Thus, 40% of the symptomatic females suffered at least once from ALF. In the entire cohort, 15/29 cases suffered from ALF (52%), and 6/29 (21%) from recurrent episodes. In none of the patients, there was any evidence for a viral infection or other concomitant illness as cause of elevated transaminases, and testing for viruses remained negative although this was not performed systematically.

### Ammonia, coagulation, response to vitamin K and liver function tests in patients

Table 2 shows laboratory data of the 15 patients with ALF that was present already at the initial crisis when the diagnosis of OTCD was made (except patient 14.2 who was diagnosed due to her affected brother).

**Table 2:** Laboratory values of OTCD patients suffering from acute liver failure.

*No*.	*Sex*	*RALF*^*a*^	*INR Ref*. *<1*.*2*	*Quick (%) Ref*. *>70*	*Albumin (g/L) Ref*. *34–42*	*Ammonia (μmol/L) Ref*. *12–48*	*ALAT (U/L) Ref*. *<33*	*ASAT (U/L) Ref*. *<50*
1	f	no	n.a.	15	n.a.	235	1900	2900
3	f	no	4.8	13	40	207	1615	1148
4.1	m	yes	2.7	n.a.	n.a.	n.a.	n.a.	n.a.
6.1	m	yes	2.5	31	34	868	35	25
8.1	m	n/a	2.2	34	n.a.	2390	23	86
9	f	no	1.5	47	45	325	95	54
11	f	no	n.a.^**b**^	n.a.^**b**^	n.a.	300	1278	411
14.1	m	yes	2.2	30	36	73	33	46
14.2	f	yes	1.7	39	35	37	19	35
16.1	m	no	1.5	43	n.a.	276	152	57
17.1	m	n/a	n.a.	6	n.a.	708	n.a.	n.a.
18	m	n/a	2.4	32	31	1821	32	82
19.1	m	yes	n.a.	20	n.a.	114	2837	1986
20	f	yes	4.2	16	28	354	739	867
21.1	m	n/a	3.0	23	n.a.	1650	13	56

^**a**^RALF, recurrent episodes of acute liver failure; n/a = not applicable, because these patients deceased during the first metabolic crisis in the neonatal period and thus could not experience RALF

^**b**^In this patient neither value for INR nor Quick was available, however, a highly pathological value for the prothrombin time

ALAT, alanine aminotransferase; ASAT, aspartate aminotransferase; Ref., reference level

In patients suffering from ALF, INR values ranged from 1.5 to 4.8 (normal < 1.2), Quick values from 6 to 47% (normal > 70), and ammonia levels from 37 to 1821 μmol/L (normal 12–48; in the neonatal period < 100).

Correlation studies provided strong evidence for a positive correlation between plasma ammonia levels and INR values in the patient cohort suffering from ALF (n = 11 [cases No. 1; 3; 4.1; 6.1; 9; 11; 14.1; 14.2; 16.1; 19.1 and 20] as expressed by a Pearson coefficient ranging from 0.12 to 0.99 with a median of 0.76, which was significant in 6/11 patients. In contrast, in patients not affected by ALF (n = 7 [cases No. 2; 6.2; 7.1; 8.2; 10; 12 and 16.2], there was no significant correlation between plasma ammonia levels and INR values in any of these patients and Pearson coefficient ranged from -0.53 to 0.85 with a median of 0.06. The positive correlation of hyperammonemia and elevated INR values in patients with ALF is exemplified for patient 20 (Table 3) who suffered from two episodes of ALF within 2 months.

**Table 3:** Association of coagulopathy and acute liver failure with hyperammonemia.

*Laboratory parameters*	*INR*	*Quick (%)*	*aPTT*^*a*^ *(sec*.*)*	*Clotting factors (%)*		*Albumin (g/L)*	*Ammonia (μmol/L)*
				*V*^*b*^	*VII*^*b*^		
***Normal Range***	<1.2	>70	<40	>70	>60	34–42	12–48
***At diagnosis***	2.3	30	42	43	14	30	498
***In remission state***	0.9	>110	n.d.	131	115	33	40
***At metabolic crisis***	4.2	16	55	24	2	28	354

Table 3 illustrates two episodes of acute liver failure, which occurred within 2 months in patient 20. While INR markedly increased and levels of factor VII (plasma half-life: 5 hours) and factor V (plasma half-life: 15 hours) decreased during hyperammonemia, all laboratory parameters normalized between events. Albumin (plasma half-life 20 days) remained in the (lower) normal range during hyperammonemic episodes.

^**a**^aPTT, activated partial thromboplastin time

^**b**^Clotting factors V and VII.

From our entire patient cohort, only siblings 14.1 and 14.2, both had pathological INR values in the presence of only mildly elevated or even normal plasma ammonia levels and both displayed only mild neurological symptoms compatible with a remission state of disease on several routine outpatient controls. In all other patients, pathological INR values exclusively occurred during hyperammonemia. In several patients, thorough analysis of individual pro- and anticoagulant factors were performed, revealing reduced functional levels of the vitamin K-dependent factors (FII, VII, IX, X, protein C and S) as well as reduced levels of the non-vitamin K-dependent factor V (as depicted in Table 4 for patient 14.1). As anticipated, neither the oral application of 5 mg vitamin K, nor an additional intravenous (i.v.) dose of 5 mg vitamin K led to an increase of the vitamin-K-dependent factors, thus virtually excluding a vitamin K deficiency (Table 4). Hemostatic factors with the shortest plasma half-lives such as factor VII (5 hours) and protein C (7 hours) showed the most pronounced reduction of plasma levels, whereas e.g. factor II with a half-life of 65 hours remained in the subnormal or low normal range. Although not systematically assessed in our patient cohort, in none of the patients we observed a response to vitamin K.

**Table 4:** Unresponsiveness to vitamin K in coagulopathy occurring in OTCD.

*Laboratory parameters*	*INR*	*Quick (%)*	*aPTT (sec)*	*Fibrinogen (g/L)*	*Factor V (%)*	*Vitamin K-dependent hemostatic factors (%)*					
						*II*	*VII*	*IX*	*X*	*Prot*. *C*	*Prot*. *S*
***Normal Range***	<1.2	>70	25–36	1.75–3.75	78–153	68–140	70–139	79–138	78–144	71–165	75–141
***outpatient visit***	2.2	30	47.1	2.3	73	65	12	n.a.	48	n.a.	n.a.
***2d after oral intake of 5 mg vit*. *K***	2.2	31	41.1	2.4	61	73	11	26	49	11	69
***before i*.*v*. *injection of 5 mg vit*. *K***	1.3	61	35.6	2.6	86	104	45	43	85	27	73
***2d after i*.*v*. *injection of 5 mg vit*. *K***	1.5	46	36.5	2.4	85	97	27	37	80	23	78
***Plasma half-lives***	*** ***	*** ***	*** ***	***100h***	***15h***	***65h***	***5h***	***25h***	***40h***	***7h***	***43h***

Table 4 shows laboratory evaluation of coagulation parameters including individual pro- and anticoagulant factors in patient 14.1 during an outpatient routine control, 2 days (2d) after oral intake of 5 mg vitamin K (vit. K), and immediately before and 2d after intravenous (i.v.) application of 5 mg vit. K. Neither of the two vit. K applications led to an increase of vitamin K-dependent factors (FII, VII, IX, X, protein C and S). Moreover, also the non-vitamin-K-dependent factor V was reduced in the first two blood examinations, thus suggesting an impaired synthetic function of the liver as reason for diminished plasmatic coagulation factors and not a vitamin K deficiency.

Taken together these laboratory data point towards a reduced hepatic synthesis of coagulation factors, which occurs intermittently with (near) normalization between exacerbations.

**Fig 3:**
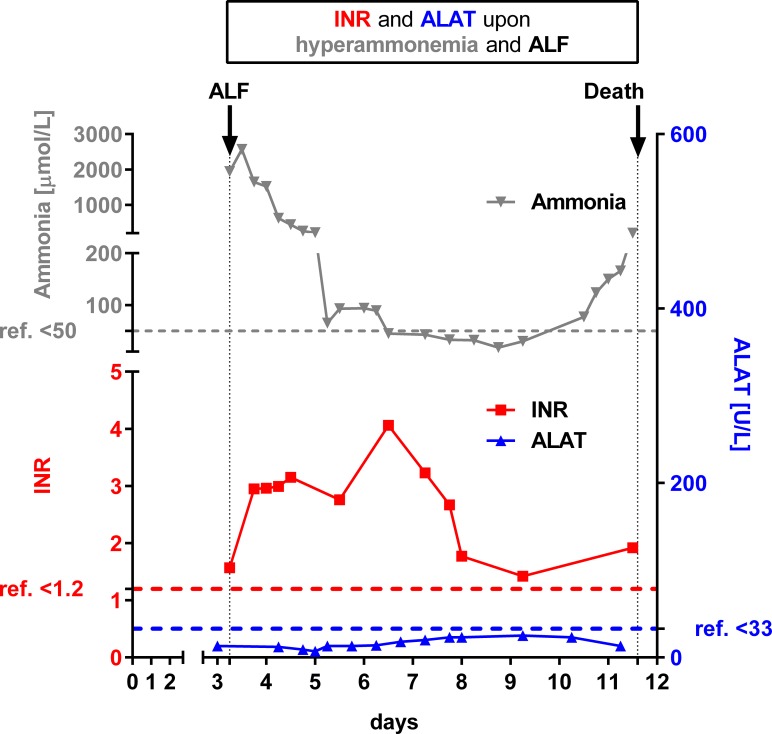
INR is a sensitive parameter of ammonia-related liver dysfunction. Fig 3 illustrates laboratory values from patient 21.1 with neonatal onset of OTCD causing acute liver failure (ALF) and fatal disease course. Concomitant with massive hyperammonemia (max. 2569 μmol/L; upper left y-axis in grey colour), INR was drastically elevated (max. 4.1; lower left y-axis in red colour) while both liver transaminases (here only ALAT is shown; max. 25 U/L) remained normal. After suspicion of OTCD, this patient immediately received specific treatment including hemodialysis resulting in rapid normalization of plasma ammonia levels after two days of treatment. However, INR remained elevated despite application of fresh frozen plasma and the patient deceased from multiorgan failure.

As listed in Table 2, liver transaminases remained normal in some patients even during severe liver involvement with massively elevated INR values and hyperammonemia. Levels of liver transaminases varied among patients and ranged from (close to) normal to highly elevated. As illustrated in patient 21.1 (Fig 3), INR increased early during metabolic decompensation, while liver transaminases remained normal during the entire episode. It is however likely that a prolonged hepatocyte dysfunction in some patients eventually leads to hepatic cell death thereby causing elevated liver transaminases.

### Experimental evidence for ammonia-induced impairment of protein synthesis

To validate the hypothesis that ammonia causes decreased hepatic protein synthesis, albumin production and secretion were investigated in isolated human primary hepatocytes upon NH_4_Cl treatment. While plasma albumin levels in patients commonly remain in the (low to) normal range during short periods of hyperammonemia due to its long plasma half-life (Table 3), in short-term cell culture studies of hyperammonemia, the suspected negative effect of NH_4_Cl on the production rate of albumin can be quantified in the cell culture supernatant. Albumin was chosen because it is robustly secreted and readily detectable in cell culture supernatant of primary hepatocytes, whereas several coagulation factors are difficult to assess upon short-term experiments due to very low concentrations [36].

**Fig 4:**
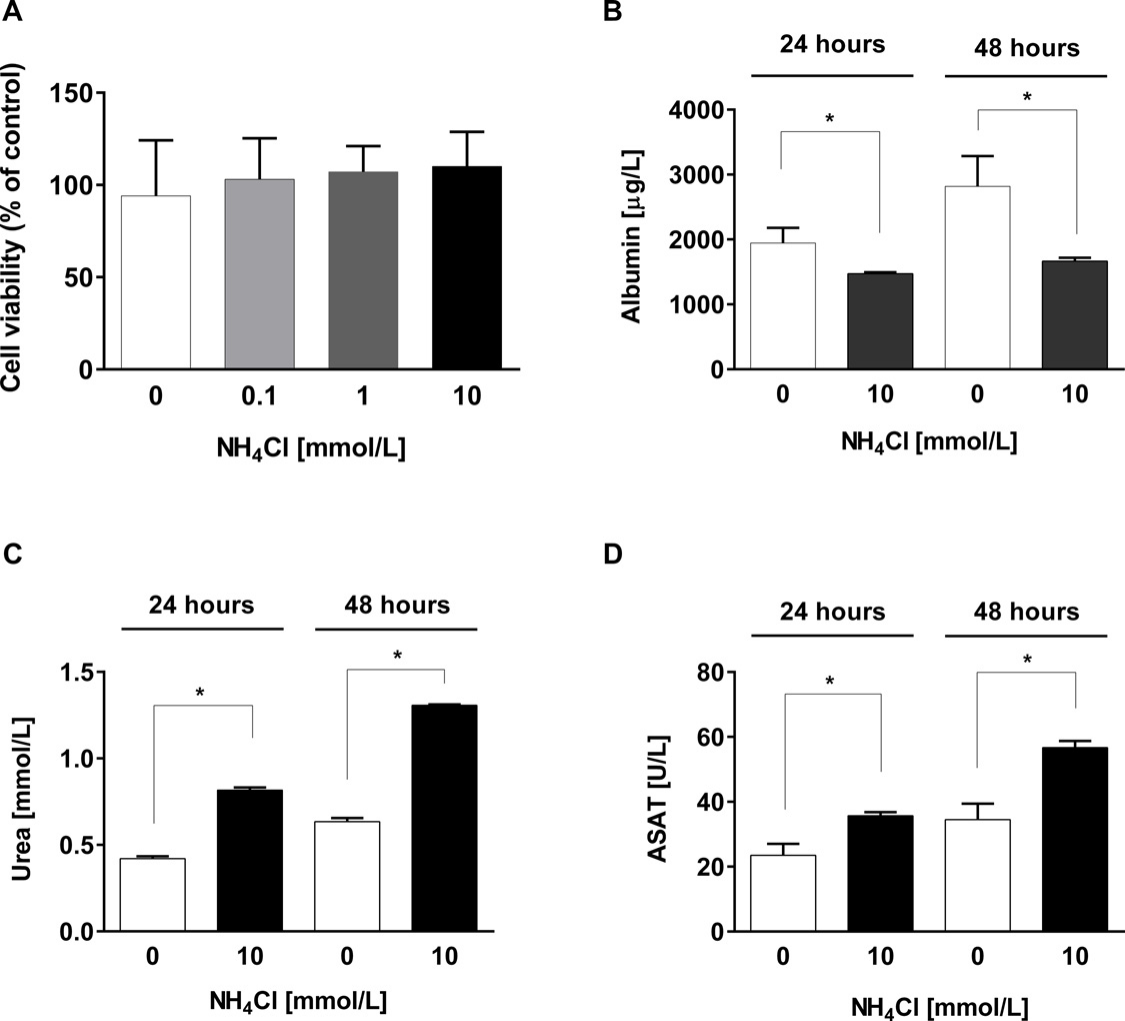
Effects of NH_4_Cl exposure on human primary hepatocytes. Following treatment with NH_4_Cl, several parameters were determined in cell culture supernatant: **A,** Cell viability showed no significant differences between various treatment groups (0; 0.1; 1; 10 mM NH_4_Cl for 24 h) compared to non-treated control cells as assessed by MTT assay. **B,** Albumin secretion was quantified by ELISA. While control cells secreted 1944 ± 235 μg/L albumin in 24 hours, cells treated with 10 mM NH_4_Cl secreted only 1471 ± 25 μg/L and after 48 hours controls secreted 2820 ± 464 μg/L versus 1666 ± 53 μg/L in treated cells. **C,** Quantification of urea: while in untreated hepatocytes urea in supernatant increases slightly from 0.42 mmol/L at 24 h to 0.64 mmol/L at 48 h, in NH_4_Cl-treated cells urea production increases from 0.82 mmol/L to 1.31 mmol/L, respectively. **D,** Quantification of ASAT reveals a potential negative effect of ammonia on mitochondrial integrity.

First, we determined cell viability to exclude (unspecific) cell death under different NH_4_Cl concentrations (0; 0.1; 1; 10 mM). Cell viability was not affected by treatment and remained unchanged to controls (Fig 4A), confirming previous reports [37]. Albumin secretion revealed a significant (*p < 0.05, Student’s T-test) decrease upon treatment with 10 mM NH_4_Cl after 24 and 48 hours compared to controls (Fig 4B). Urea production was increased during treatment with NH_4_Cl indicating a functioning urea cycle in these cells (Fig 4C). Quantification of ASAT revealed a potential negative effect of ammonia on mitochondrial integrity as it increases over time in the treated cells (Fig 4D).

## Discussion

The effects of ammonia toxicity on the central nervous system have been intensely studied using several *in vitro* and *in vivo* models that suggested ammonia toxicity to the developing brain by alteration of amino acid and neurotransmitter pathways, by interference with the tricarboxylic acid cycle, ATP production and concomitantly brain energy metabolism [38]. In contrast, little is known about the effects of elevated ammonia on the hepatic system. A prime example of the effect of hyperammonemia on liver function is the group of patients with primary acute hyperammonemia who develop ALF and concomitant coagulopathy as reported previously [5–9], and as described here. Although previous reports have described UCD patients with markedly increased INR/PT values during metabolic crises, thus reflecting ALF [6, 11], the underlying pathophysiology is unclear. Uncovering the molecular mechanisms leading to ALF may help tailor treatment decisions including the development of consensus criteria for liver transplantation.

Analysis of our OTCD cohort revealed ALF as a very frequent complication with 100% of male patients and 40% of symptomatic females being affected at least once during disease course. This gender difference is most likely explained by the X-chromosomal inheritance of OTCD, rendering (especially neonatal) male patients at a higher risk of metabolic decompensation with hyperammonemia and concomitant ALF. Our epidemiological data confirm the so far only systematic clinical study of liver involvement in OTCD patients [6], and underlines that hepatic complications of UCDs may be an underrecognized condition. Affected neonates often suffer from multiorgan failure and may not survive the initial metabolic crisis. Concomitant liver dysfunction may not be considered as a specific complication of OTCD, thus not receiving enough attention, particularly during the emergency management of acute hyperammonemia. However, ALF may be a trigger of multiorgan failure and a rapid and targeted intervention could prevent, at least in some patients, an often fatal disease course. Moreover, ALF may be underrecognized because specific clinical symptoms such as severe bleeding may be missing. Although several of our patients suffered from recurrent (laboratory) coagulopathy with massively elevated INR values, bleeding complications did not belong to the characteristic complications. The reason for the low incidence of bleeding may be the simultaneous impairment of procoagulant and anticoagulant hemostatic factors (as demonstrated in Table 4 for patient 14.1), thereby mitigating the risk for hemorrhagic events [39, 40].

The main laboratory finding in our cohort is the close association of elevated INR values with high plasma ammonia levels. This suggests a causative role of hyperammonemia in impairing hepatic protein synthesis thus leading to a rapid reduction of short-lived plasma coagulation factors. In fact, all patients in our cohort for whom data were available showed a rapid decrease of coagulation factor VII in plasma and concomitantly an increase of INR consistent with ALF as the cause of the coagulopathy. At the same time, in most neonatal patients liver transaminases remained normal despite severe liver involvement. Taken together, these laboratory data suggest an ammonia-induced inhibition of hepatic protein synthesis rather than (unspecific) cell death as underlying mechanism of hyperammonemia-induced ALF.

In our cohort, we have two siblings (14.1 and 14.2) who suffered from RALF in whom we observed pathological INR values even in the absence of hyperammonemia. We cannot conclude whether in certain patients, even slight (or intermittent) elevations of ammonia are sufficient to cause ALF or whether there are other potential triggers causing ALF. It was previously reported by others that coagulopathy rarely occurs in OTCD patients even in remission state in absence of relevant hyperammonemia [10]. The authors speculated that a potential vitamin K deficiency could be the reason for coagulopathy in affected patients. However, analysis of serum des-γ-carboxyprothrombin (PIVKA-II, a marker of vitamin K deficiency) was normal in 3 of 4 of their analyzed patients. In line with that, in none of our patients (as illustrated in Table 4 for patient 14.1) we found a vitamin K response.

To further explore our hypothesis of ammonia-induced inhibition of hepatic protein synthesis, we performed *in vitro* experiments using isolated primary hepatocytes from a non-affected donor. First, cell culture studies showed a significantly reduced production rate of albumin (the most abundant secreted hepatic protein) upon NH_4_Cl treatment. Next, when applying up to 10 mmol/L NH_4_Cl we did not detect cytotoxicity but found a moderate increase of ASAT, thus indicating a negative effect on mitochondrial integrity. This is in line with recently published data of an inhibitory effect of ammonia on mitochondrial integrity in hepatocytes *in vitro* when high concentrations of NH_4_Cl (20 mmol/L) induced an intrinsic Ca^2+^-independent apoptosis pathway [18].

Although primary human hepatocytes are a useful tool to study effects of ammonia toxicity on a molecular level, there are certain limitations. One major drawback of primary hepatocytes is their lack of availability. Besides that, despite the enormous replication potential of hepatocytes *in vivo* they cannot serve as culture for long-term studies *in vitro* [41]. Moreover, while we aim to investigate pathophysiological differences between “healthy” human primary hepatocytes and UCD patient-derived hepatocytes in an *in vitro* system, access to UCD patient hepatocytes is utmost challenging and very difficult to plan in advance.

In summary, ALF is a common complication of acute hyperammonemia in patients with OTCD and was also reported in other UCDs such as citrullinemia type 1 [5, 13]. In an attempt to better understand the underlying mechanisms, we found strong evidence that ammonia displays a direct toxic role on hepatic protein synthesis. However, while the association of hyperammonemia and ALF is clear, the exact pathophysiological sequence may be even more complicated although this is rather speculative. As a clinical consequence, we suggest adding INR test in routine care of patients with liver dysfunction early during their work-up. Building up on our findings, further studies of ALF in other UCDs and likewise in other conditions of (secondary) hyperammonemia will shed light on the involved pathophysiological mechanisms and will aid the decision on whether or not to perform urgent liver transplantation in ALF and/or other therapeutic strategies.
